# How Healthy Aging and Contact With Children Are Associated With Satisfaction in Middle-Aged and Older Parents in China: A Mediation Analysis

**DOI:** 10.3389/fpubh.2022.836558

**Published:** 2022-03-14

**Authors:** Jiangyun Chen, Yixin Zeng, Wenjun He, Jiao Yang, Dong Xu, Haomiao Li

**Affiliations:** ^1^Center for World Health Organization (WHO) Studies and Department of Health Management, School of Health Management of Southern Medical University, Guangzhou, China; ^2^ACACIA Labs of Southern Medical University Institute for Global Health (SIGHT) and Dermatology Hospital, Southern Medical University (SMU), Guangzhou, China; ^3^Institute for Health Management, Southern Medical University, Guangzhou, China; ^4^School of Political Science and Public Administration, Wuhan University, Wuhan, China

**Keywords:** healthy aging, family context, older parents, contact with children, satisfaction, Chinese

## Abstract

**Objectives:**

This study aims to examine the mediation role of satisfaction with children on the association between contact with children (CCT) and healthy aging among middle-aged and older parents in China.

**Methods:**

Data from 9,575 parents over 45 years old were obtained from the 2018 China Health and Retirement Longitudinal Survey. A multinomial logistic regression model was applied to measure the association between contact, satisfaction, and healthy aging with potential confounders controlled. We used the Sobel–Goodman Mediation test to analyze the mediation role of satisfaction on the association between types of CCT and healthy aging.

**Results:**

Parents with contact with adult children had higher satisfaction with children [for contact weekly (satisfied/unsatisfied): relative risk ratio (*RRR*) = 2.44, *CI* = 1.92–3.10] and higher healthy aging [for contact weekly (Q5/Q1): *RRR* = 1.41, *CI* = 1.13–1.77]. Satisfaction was strongly related to healthy aging [for satisfied (Q5/Q1): *RRR* = 3.44, *CI* = 2.14–5.51], and mediated 19.05% of healthy aging for weekly contact (Sobel test *z* = 4.338; indirect role = 0.014, *CI* = 0.011–0.018; direct role = 0.061, *CI* = 0.029–0.094). Subgroup analysis further revealed that satisfaction with contact played a partial mediating role between monthly contact and healthy aging in female and rural groups.

**Conclusions:**

Monthly CCT is more appropriate for older parents. Satisfaction with children in older parents seems to act as a significant and partial mediator of the relationship between contact and healthy aging. The contribution of satisfaction to healthy aging could be important to be considered and promoted in women and rural older parents, independent of CCT.

## Introduction

China's population is rapidly aging; by 2050, there will be nearly 400 million Chinese citizens aged 65 years old or older, 150 million of whom will be 80 years old or older (1). By 2065, the old-age dependency ratio will more than triple (2). Compared with developed countries, China's economic development level is relatively backward in terms of the aging of its society; one of its prominent characteristics is “getting old before getting rich” (3). Demographic changes, economic reforms, and modernization in China have been remarkable, but the attendant social and work pressures have weakened the ability of families to support older family members. These circumstances may challenge China's response to population aging.

As the conventional central family model is increasingly uncommon (4), one- and two-generation family households are more common. Support from adult children is one of the most important family resources related to parents' ability to adapt to functional decline (5). These resources are the material and spiritual support needed to maintain essential family functions and respond to stressful events and crises (6, 7). Family systems theory (8) further reveals the interrelatedness of family members. Life satisfaction refers to the cognitive-judgmental aspects of one's life experience (9), whereas satisfaction with children refers to the subjective feelings and evaluations of interactions and relationships with their children. Moreover, the family members' communication competence, time spent conversing among themselves, and their personalities all play an important role in explaining their satisfaction with their family relationship (10–12). As interactions with adult children seem to predict satisfaction with family relationships and adaptation to functional decline, there is a strong need to examine whether health aging in middle-aged and older parents could be managed more effectively under the influence of strong contact with adult children as well as satisfaction with their relationships with their children.

Healthy aging is defined by the WHO as the process of developing and maintaining the functional ability that enables wellbeing in older age. Healthy aging is a holistic view that encompasses both biological and medical aspects of aging, as well as subjective experiences and meanings, and emphasizes functional definitions of autonomy, participation, and wellbeing (13). As healthy aging is a comprehensive concept it is affected by many factors, such as demographic background, socio-economic status, health behaviors, and social interaction (14–16), all of which must be measured.

The role of adult children has become an important aspect of assessing the health status of the parents. Consistently low levels of positive support and an increase in negativity from children have been associated with increased mortality (17). Anticipated support from children is related to older parents' better self-rated health and fewer depressive symptoms (18). Child-related factors (number of children, proximity, and visits) were strongly associated with mental health in parents aged 80 and older after adjusting for socioeconomic factors, social support, health behaviors, and health status (19). In the context of China's current family structure, where many children do not live with their parents (20), contact with children (CCT) could be more reflective of the children's support and care for their parents than living with them. To date, research is inconclusive regarding how CCT affects healthy aging in older parents.

Filial piety is a key virtue in Chinese culture and was considered the bedrock of the social care and health system that protected and supported older people in China (21). The relationship between the parents and their adult children is the most common source of intergenerational support (22), and interaction with children is the manifestation of the parent-child relationship in daily life. There are many studies that reveal the effects of parent-child relationships on children (23–27), but few studies have examined how these relationships may have a possible association with the health of the parents (28). Parents' metabolic health may, in part, be influenced by aspects of the parent-child relationship (29), and parents tend to suffer from depression as there are decreases in the frequency of seeing their adult children and their satisfaction with the parent-child relationship (30). One study (31) suggests that increased frequency of child-parent contact may have a positive impact on relationship satisfaction on children. Healthy aging is a highly fluid and complex issue that requires further studies examining the relationship between CCT, satisfaction with relationships, and healthy aging among middle-aged and older parents to develop theoretical and empirical understanding of this under-researched area.

### The Present Study

The role of CCT in the relationship between children satisfaction and healthy aging has not been explored in any previous studies, despite the potential role of relationship satisfaction as an efficient intervening measure that could promote healthy aging (16). This study aims to examine the mediation role of satisfaction with the relationship with children (CSF) on the association between CCT and the Healthy Aging Index (HAI) among middle-aged and older parents in China. Based on the literature review above, the following hypotheses are proposed:

Hypothesis 1. CCT improves CSF and HAI among the middle-aged and older population.
Hypothesis 2. CSF is also positively correlated with HAI. That is, CSF plays a mediation role in the association of CCT with HAI.

## Methods

### Data and Procedure

China Health and Retirement Longitudinal Study (CHARLS) national survey of wave four was conducted from August, 2018 to March, 2019. The CHARLS is a biennial/triennial survey conducted by the National School of Development of Peking University, collecting high-quality micro-data from middle-aged and older individuals aged 45 years and above in China. The individual questionnaire includes basic demographics and information on family interaction, health status and functioning, healthcare and insurance, employment, retirement, and pension, income and consumption, household assets, and so on. Details on the sampling method and questionnaire are available at the official website (URL: http://charls.pku.edu.cn/) and in many other studies. The CHARLS research team and field teams collect the data and make the data publicly available. The total sample size of the CHARLS survey in 2018 was 19,816 individual respondents. We limited our sample to parents who had at least one alive child. Parents who had missing dependent values, independent variables, and mediation variables were excluded. The final sample contained 9,575 parents.

### Healthy Aging Index

The HAI was measured using a healthy aging scale (BMRM-D-18-00329) developed by the Aging Trajectories of Health: Longitudinal Opportunities and Synergies (ATHLOS) project (32), which consisted of 16 general population longitudinal studies (33). Item response theory model was used to develop an ATHLOS scale with 41 items for measuring healthy aging (34). By drawing on existing studies, 32 items in the CHARLS database were selected for measuring HAI (see Supplementary Table 1), covering various aspects of health and aging, such as mobility, sensory skills, cognition, vitality, psychological symptoms, activities of daily living and instrumental activities of daily living, and latent trait scores were estimated. To provide a straightforward interpretation of the results, the estimated latent trait scores were rescaled to a range from 0 (least healthy) to 100 (most healthy) (35). We determined levels of healthy aging by quintiles of the HAI. Parents were categorized into five groups: Q1 (“very low” of HAI), Q2 (“low”), Q3 (“moderate”), Q4 (“high”), and Q5 (“very high”) (36).

### Satisfaction With Children (CSF)

Satisfaction is a new variable added in the fourth round of the survey. Parents were asked, “how satisfied are you with your relationship with your children.” The item was rated from 1 (completely satisfied) to 5 (completely dissatisfied). Based on this, CSF was categorized into 3 groups: 1 = satisfied (completely satisfied, very satisfied); 2 = somewhat satisfied; 3 = not satisfied (not very satisfied, not at all satisfied).

### Contact With Children (CCT)

The parents were asked how often do they have contact, in person or by phone, with each of their adult children. Based on this, we categorized the CCT into 4 independent variables: daily CCT, weekly CCT, monthly CCT, and two times-yearly CCT (see Supplementary Table 2).

### Control Variables

Covariates were selected on the basis of their association with the independent variable and their impact on the change of the association between the independent and dependent variables. Age and gender were included as fixed covariates to be adjusted. The other covariates were included as potential confounders in the final models if they changed the estimates of CCT/CSF on HAI score by more than 10% or were significantly associated with HAI score. The following covariates were selected based on established associations and/or plausible biological relations and tested: hukou status (indicates the respondent's hukou place and is a special identifier in China, hukou status affects many aspects of life in China, such as buying a house, buying a car, children's school enrollment, and other welfare), residence, education levels, public health insurance coverage, current work status, smoking, alcohol intake, self-report of health, chronic condition, household per capita consumption, whether they live near children, number of living children, gave money to children, and received money from children. The associations of each confounder with HAI score are detailed in Supplementary Tables 3–5.

### Data Analysis

Characteristics of parents across the categories of weekly CCT (yes, no, and total) are presented as the mean ± *SD* and the frequency (%) for continuous and categorical variables, respectively. HAI groups (Q1–Q5) across the categories of CCT (yes and no) and CSF (satisfied, somewhat satisfied, and not satisfied) are presented as the frequency (%). Characteristic differences were examined using Student's *t*-test for continuous variables and the chi-squared test for categorical variables. Multinomial logistic regression models (MLRM) were employed to estimate the association between CCT, CSF, and HAI. Relative risk ratio (RRR) with 95% CIs was calculated after adjusting for age, gender, hukou status, residence, education levels, public health insurance cover, current work status, smoking, alcohol intake, self-report of health, chronic condition, household per capita consumption, live near children, number of living children, transfer to children, and transfer from children. We analyzed the mediation role of CSF on the association of CCT (daily CCT, weekly CCT, monthly CCT, and twice a year CCT) with HAI through Sobel–Goodman Mediation Test, with all the selected covariates controlled.

The *p*-values were 2-sided, and an alpha level of 0.05 was used to define statistical significance. Data were analyzed using Stata (version 16) and R version 3.6.3 (R Foundation for Statistical Computing, Vienna, Austria).

## Results

### Parents' Characteristics

The mean age was (65.17 ± 8.39) years old. Approximately, 52.32% of the parents had three or more alive children. Most (84.31%) of subjects contacted their children weekly, but only 49.22% live together with their children. Approximately, 45.94% of them were male and 83.72% of them were married. The vast majority (89.35%) of them was graduated from junior high school and below. Approximately, 66.38% of them were from rural areas and 61.34% had a job. Approximately, 96.60% of them were insured and 68.52% of them seldom drank. The prevalence of smoking was 25.93%. Four-fifth of the subjects had one or more chronic diseases. The rate of subjects satisfied with their relationship with their children was 57.46%.

Those who had weekly contact with their children were more likely to have non-agricultural hukou and be from urban areas, have higher-level education and more household consumption, and have more financial interaction with their children. It indicated that lower socioeconomic levels hindered the CCT. Also, CSF was significantly higher in parents who had weekly CCT (χ^2^ = 94.991, *p* < 0.001) (as shown in Supplementary Table 6). HAI was higher in subjects who had CCT (daily CCT, weekly CCT, and monthly CCT) and who had higher satisfaction. The results are shown in Table 1.

**Table 1:** Distribution of healthy aging across contact with children (CCT) and satisfaction in parents aged 45 and older, [*N* (%)].

	**Total**	**HAI**					**χ^2^**	***P-*value**
		**Quintile 1**	**Quintile 2**	**Quintile 3**	**Quintile 4**	**Quintile 5**		
**Daily CCT**							**10.665**	**0.031**
No	5,589 (58.37%)	1,059 (55.30%)	1,116 (58.28%)	1,151 (60.10%)	1,128 (58.90%)	1,135 (59.27%)		
Yes	3,986 (41.63%)	856 (44.70%)	799 (41.72%)	764 (39.90%)	787 (41.10%)	780 (40.73%)		
**Weekly CCT**							**32.293**	**<0.001**
No	1,502 (15.69%)	354 (18.49%)	300 (15.67%)	324 (16.92%)	292 (15.25%)	232 (12.11%)		
Yes	8,073 (84.31%)	1,561 (81.51%)	1,615 (84.33%)	1,591 (83.08%)	1,623 (84.75%)	1,683 (87.89%)		
**Monthly CCT**							**29.001**	**<0.001**
No	230 (2.40%)	73 (3.81%)	56 (2.92%)	32 (1.67%)	37 (1.93%)	32 (1.67%)		
Yes	9,345 (97.60%)	1,842 (96.19%)	1,859 (97.08%)	1,883 (98.33%)	1,878 (98.07%)	1,883 (98.33%)		
**Twice a year CCT**							**4.085**	**0.395**
No	78 (0.81%)	18 (0.94%)	21 (1.10%)	11 (0.57%)	15 (0.78%)	13 (0.68%)		
Yes	9,497 (99.19%)	1,897 (99.06%)	1,894 (98.90%)	1,904 (99.43%)	1,900 (99.22%)	1,902 (99.32%)		
**CSF**							**166.598**	**<0.001**
Not satisfied	422 (4.41%)	158 (8.25%)	99 (5.17%)	80 (4.18%)	54 (2.82%)	31 (1.62%)		
Somewhat satisfied	3,651 (38.13%)	762 (39.79%)	783 (40.89%)	741 (38.69%)	734 (38.33%)	631 (32.95%)		
Satisfied	5,502 (57.46%)	995 (51.96%)	1,033 (53.94%)	1,094 (57.13%)	1,127 (58.85%)	1,253 (65.43%)		

*N = 9,675. CSF, Satisfaction with the relationship with children; CCT, contact with children; HAI, healthy aging index. Differential distribution of health aging by contact and satisfaction were analyzed using χ^2^. Values are bolded if they achieved statistical significance at p ≤ 0.05*.

### Association Between CCT, CSF, and HAI

A multinomial logistic regression model was conducted to investigate whether CCT affected the CSF and HAI adjusting for potential confounders. The null hypothesis was βi = 0. Parents who had CCT had higher satisfaction with their relationships [for weekly CCT (satisfied/unsatisfied): *RRR* =2.44, *p* < 0.001, *CI* = 1.92–3.10], and HAI [for weekly CCT (Q5/Q1): *RRR* = 1.41, *p* = 0.003, *CI* = 1.13–1.77] as shown in Table 2. Table 3 shows that parents who were satisfied with the relationship with their children had higher HAI [for satisfied (Q5/Q1): *RRR* = 3.44, *p* < 0.001, *CI* = 2.14–5.51].

**Table 2:** Multinomial logistic regression analysis for CCT associated with satisfaction and healthy aging in parents aged 45 and older, [relative risk ratio (RRR) (95% *CI*)].

**CCT**	**CSF**		**HAI**			
	**Somehow satisfied**	**Satisfied**	**Quintile 2**	**Quintile 3**	**Quintile 4**	**Quintile 5**
Daily CCT	0.94 (0.76, 1.17)	1.07 (0.86, 1.32)	0.99 (0.86, 1.14)	0.94 (0.82, 1.09)	1.06 (0.91, 1.23)	1.08 (0.91, 1.27)
Weekly CCT	**1.66 (1.31, 2.11)** ^***^	**2.44 (1.92, 3.10)** ^***^	**1.21 (1.01, 1.45)** ^*^	1.02 (0.85, 1.24)	1.15 (0.94, 1.41)	**1.41 (1.13, 1.77**)^**^
Monthly CCT	**3.33 (2.18, 5.08)** ^***^	**4.21 (2.77, 6.39)** ^***^	1.28 (0.87, 1.89)	**2.22 (1.39, 3.55)** ^***^	**2.05 (1.27, 3.31)** ^**^	**2.47 (1.44, 4.23)** ^***^
Twice a year CCT	**2.87 (1.40, 5.87)** ^**^	**4.37 (2.12, 9.01)** ^***^	1.06 (0.53, 2.12)	2.19 (0.94, 5.11)	1.76 (0.78, 3.96)	**2.57 (1.02, 6.48)** ^*^

*N = 9,675. RRR, relative risk ratio; 95% CI, 95% confidence intervals; CSF, Satisfaction with the relationship with children; CCT, contact with children; HAI, healthy aging Index. Pseudo R^2^-values in adjusted models for age, gender, hukou status, residence, education levels, public health insurance cover, current work status, smoking, alcohol intake, self-report of health, chronic condition, household per capita consumption, live near children, number of alive children, gave money to children, and received money from children. Values are bolded if they achieved statistical significance at p ≤ 0.05*.

*“Not satisfied” and “Quintile 1” (“very low” of HAI) were chosen as the reference group to predict the probability of higher CSF and higher HAI, with CCT as a protective factor at RRR > 1*.

* *p < 0.05,*

** *p < 0.01,*

*** *p < 0.001*.

**Table 3:** Multinomial logistic regression analysis for satisfaction associated with healthy aging in parents aged 45 and older [RRR (95% *CI*)].

**CSF**	**HAI**			
	**Quintile 2**	**Quintile 3**	**Quintile 4**	**Quintile 5**
Somewhat satisfied	**1.42** (1.07, 1.90)^*^	**1.53** (1.11, 2.10)^**^	**2.04** (1.40, 2.97)^***^	**2.72** (1.69, 4.39)^***^
Satisfied	**1.37** (1.03, 1.82)^*^	**1.60** (1.17, 2.20)^**^	**2.18** (1.50, 3.16)^***^	**3.44** (2.14, 5.51)^***^

*RRR, relative risk ratio; 95% CI, 95% confidence intervals; CSF, Satisfaction with the relationship with children; HAI, healthy aging index. Pseudo R^2^-values in adjusted models for age, gender, hukou status, residence, education levels, public health insurance cover, current work status, smoking, alcohol intake, self-report of health, chronic condition, household per capita consumption, live near children, number of alive children, transfer gave money to children, received money from children, and weekly contact. Values are bolded if they achieved statistical significance at p ≤ 0.05*.

*“Quintile 1” (“very low” of HAI) was chosen as the reference group to predict the probability of higher HAI, with CSF as a protective factor at RRR > 1*.

* *p < 0.05,*

** *p < 0.01,*

*** *p < 0.001*.

Two subgroup analyses of gender and residence were performed through MLRM (see Supplementary Table 7). Weekly, monthly, and twice a year CCT were positively associated with HAI in the rural subgroup, whereas the association between different frequencies of CCT and HAI was not significant in the urban subgroup. Weekly CCT was positively associated with HAI in the man subgroup, whereas monthly CCT was positively associated with HAI in the woman subgroup. Weekly and monthly CCT were positively associated with CSF in all subgroups, and twice a year CCT was positively associated with CSF in men and rural groups. Supplementary Table 8 shows that CSF was positively associated with HAI in all subgroups.

### Mediating Effect of CSF on the Relationship Between CCT and HAI

Weekly, monthly, and twice a year CCT were positively associated with HAI among middle-aged and older parents. Mediation analysis, such as CSF, revealed that the association between those three frequencies of CCT and HAI was mediated *via* CSF. CSF played a partial mediating role between CCT and HAI in this study, and the role of this mediation accounted for from 7.42% (monthly CCT) to 19.05% (weekly CCT) with potential confounders adjusted (see Supplementary Table 9).

**Figure 1:**
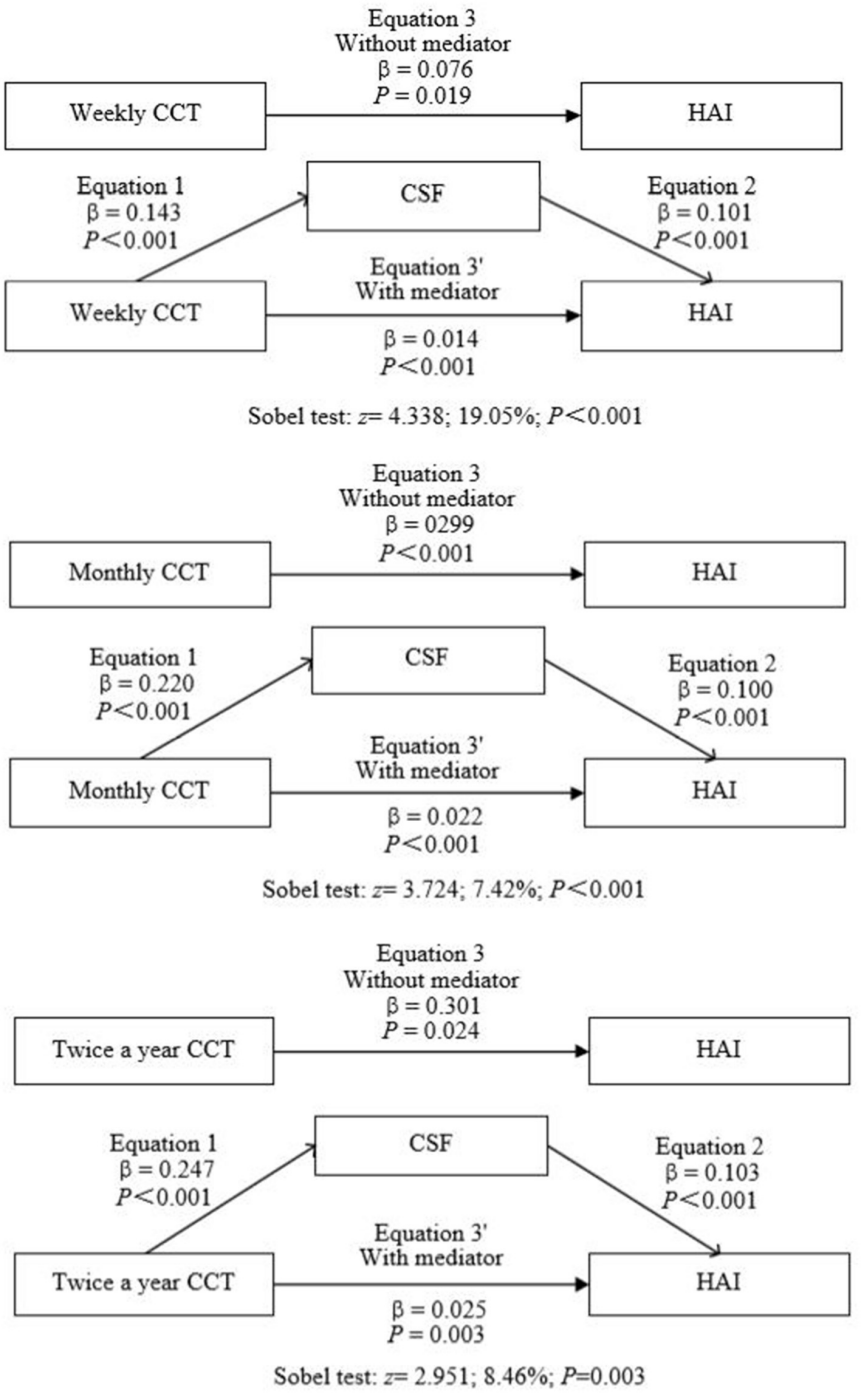
Mediation analysis: contribution of child contact to healthy aging through parental satisfaction age 45 and over. CSF, Satisfaction with the relationship with children; HAI, healthy aging. The Sobel test was used to test the hypothesis that the indirect role was equal to 0, adjusting for potential confounders (age, gender, hukou status, residence, education levels, public health insurance cover, current work status, smoking, alcohol intake, self-report of health, chronic condition, consumption, live near children, number of alive children, gave money to children, and received money from children). Values are bolded if they achieved statistical significance at *p* ≤ 0.05.

Weekly CCT was related to CSF (β = 0.143, *p* < 0.001) and related to HAI (β = 0.014, *p* < 0.001). CSF was also related to HAI (β = 0.101, *p* < 0.001). CSF mediates 19.05% of HAI for weekly CCT (*z* = 4.338; indirect role = 0.014, *CI* = 0.011–0.018; direct role = 0.061, *CI* = 0.029–0.094; *p* < 0.001). The final mediation models of the independent variable (HAI), the mediator variable (CSF), and the dependent variable (CCT) were shown in Figure 1.

Subgroup analysis of gender and residence was shown in Table 4. CSF played a partial mediating role between monthly CCT and HAI in women (*z* = 2.885; indirect role = 0.025, *CI* = 0.016–0.033; direct role = 0.371, *CI* = 0.265–0.477; *p* < 0.001), and rural (*z* = 3.093; indirect role = 0.021, *CI* = 0.015–0.028; direct role = 0.337, *CI* = 0.251–0.424; *p* < 0.001). CSF also played a partial mediating role between twice a year CCT and HAI in rural (*z* = 2.631; indirect role = 0.028, *CI* = 0.017–0.038; direct role = 0.363, *CI* = 0.210–0.516; *p* = 0.008).

**Table 4:** Gender and residence subgroup analysis of the mediation model of healthy aging vs. 45 and older parent satisfaction-mediated child exposure.

	**Indirect effect**	**Direct effect**	**Total effect**	***Z***	**Sobel *p-*value**	**Proportion of total effect that is mediated %**
**Male**						
Daily CCT	0.002	0.027	0.029	1.404	0.160	8.55
Weekly CCT	0.011^*^	0.064	0.075	2.480	0.013^*^	15.08
Monthly CCT	0.019^*^	0.165	0.184	2.271	0.023^*^	10.12
Twice a year CCT	0.030^*^	0.244	0.275	2.238	0.025^*^	11.08
Female						
Daily CCT	0.003	0.022	0.025	1.447	0.148	12.41
Weekly CCT	0.017^***^	0.060	0.078	3.610	< 0.001^**^	22.48
Monthly CCT	0.025^**^	0.371^***^	0.396^***^	2.885	0.004^**^	6.27
Twice a year CCT	0.016	0.309	0.325	1.300	0.194	4.83
Rural						
Daily CCT	0.002	−0.005	−0.003	1.308	0.191	−80.70
Weekly CCT	0.013^***^	0.054	0.067	3.519	<0.001^***^	19.99
Monthly CCT	0.021^**^	0.337^***^	0.358^***^	3.093	<0.001^***^	5.98
Twice a year CCT	0.027^**^	0.363^*^	0.390^**^	2.631	0.009^**^	7.00
Urban						
Daily CCT	0.004	0.070	0.074	1.580	0.114	5.54
Weekly CCT	0.018^**^	0.085	0.104	2.618	0.009^**^	17.71
Monthly CCT	0.027^*^	0.024	0.003	2.116	0.034^*^	949.19
Twice a year CCT	0.020	−0.043	−0.022	1.232	0.218	−91.11

*CSF, Satisfaction with the relationship with children; CCT, contact with children; HAI, healthy aging Index. Sobel-Goodman mediation test in adjusted models for age, gender, hukou status, residence, education levels, public health insurance cover, current work status, smoking, alcohol intake, self-report of health, chronic condition, household per capita consumption, live near children, number of alive children, gave money to children, and received money from children. Values are bolded if they achieved statistical significance at p ≤ 0.05*.

* *p < 0.05,*

** *p < 0.01,*

*** *p < 0.001*.

## Discussion

Since the 1980s, the household system in almost all Chinese regions has evolved from a large unitary model to a small diversified one (37). In this study, we found that only 49.22% of parents lived near their adult children. This may add weight to the impact of CCT on the CSF and HAI of the parents. Compared with parents who did not have contact with their adult children, those who had contact with their adult children (weekly, monthly, and twice a year CCT) had a higher likelihood of CSF as well as HAI. CCT directly influences HAI by supporting the parents' health and by providing assistance in a timely fashion; it also indirectly improves HAI by enhancing CSF. This finding echoes family systems theory (8), indicating the vital role of enhanced family members' interaction in family relationship satisfaction and older members' healthy aging.

### Hypothesis 1

CCT improves CSF and HAI among the middle-aged and older population, was supported. Interdependency and relation-oriented responsibilities are culturally emphasized in China (38). Older people tend to suffer from depression as the frequency of meeting and relationship satisfaction with children decreases (23), typically because psycho-social problems (such as lack of CCT) increase loneliness (39), and high levels of loneliness may signal insufficient closeness in social relationships as well as a lack of hope for the future. These factors can disrupt interpersonal wellbeing (40). Prolonged failure to contact older people in person or by phone can constitute emotional abuse of a parent by a family member (41).

### Hypothesis 2

CSF is associated with HAI, and plays a mediation role in the association of CCT with HAI, which was also supported. HAI was higher in the parents who had higher CSF. And CSF in the parents seems to act as a significant and partial mediator of the relationship between CCT and HAI. More specifically, better HAI was achieved through CSF that was improved by weekly, monthly, and twice a year CCT. CSF may be associated with both physical and mental health for the older parents (28–30). Regular contact from children is in line with traditional Chinese filial culture (21) and has a positive impact on older parents' satisfaction with their children.

Meanwhile, the results of the subgroup analysis further reveal that CSF played a partial mediating role between monthly CCT and HAI in women and rural groups, but showed no signification in male and urban groups. Moreover, twice a year CCT was associated with HAI *via* CSF in the rural group. Workforce participation helps healthy family members (especially children) cope with chronic illness in older parents aged 60 years and older, and maintain normal family functioning (42), and it is extremely common in rural China where younger people go to the city to work and send money home to their parents, and they often only return home during the Chinese New Year (43), a situation that increases the geographical barriers for rural middle-aged and older parents to connect with their adult children. This explains, in part, why the impact of CCT on HAI among rural middle-aged and older parents is more pronounced. Women are able to recognize the importance of social networks to alleviate issues of isolation and loneliness and utilize existing support networks in times of stress in their lives (44). Furthermore, Chinese women are more emotional than men, and the gender differences are larger for middle-aged and older parents than for younger ones (45). Therefore, women can more easily capture and utilize contact with their adult children as a strong family resource. More importantly, women are physically frailer and have a higher rate of disability, but have a substantially higher rate of survival at all ages compared with men (46, 47), which may add variability to HAI in women.

Study results also indicated that CCT was hindered by lower socioeconomic levels. To improve CSF and HAI, countermeasures should be suggested that can increase CCT. First of all, the focus should be on the middle-aged and older population with low CCT, and particularly, financial support should be given to middle-aged and older people in rural areas, without spouses, with few children and low economic income levels. Second, in response to the development trend of the new digital economy model (48, 49), new job opportunities, such as rural e-commerce and community group buying, should be used to attract young people to return to their hometowns for employment. Third, more diversified methods of contact should be strengthened, especially for rural residents (whose children are unlikely to visit regularly), help seniors learn to use online platforms, such as video calling, WeChat, and Jitterbug. On the one hand, we can popularize smart devices through subsidies and gifts, and conduct centralized teaching at the village and community levels, as well as teaching within the family; on the other hand, we can make age-appropriate modifications to smart devices and application software, such as increasing large fonts, louder playback, and remote assistance services. Fourth, increase television and radio publicity to create a favorable atmosphere for parent-child contact to reduce the incidence of abuse and neglect of older people (50) and draw on the framework for global mental health capacity building (51) to develop interventions for non-contact from children.

Moreover, CSF can be seen as an outward expression of the parent-child relationship, and a higher CSF implied a closer and more harmonious parent-child relationship, which was shown to have a positive impact on the health of the parents (28–30). Therefore, CSF contribution in HAI could be important and should be promoted for female and rural middle-aged and older parents independently of CCT.

#### Strength and Limitations

This study makes significant contributions to the literature by assessing the association between CCT, CSF, and HAI among middle-aged and older parents, using a nation-wide representative database. CCT seemed to be more important for women and rural middle-aged and older parents. In particular, this study appeared to provide evidence of a more appropriate frequency of CCT, in other words, monthly CCT was significant for improving CSF and HAI, while neither high frequency (contact once a week) nor low frequency (contact two times a year) was significant. This suggests that monthly CCT may be a more appropriate rhythm. This frequency of CCT may maintain a balance between intimacy and alienation and lead to better results, which is also consistent with the psychological effects of interpersonal interactions.

The results and implications of this study should be considered in light of several limitations. First, given the exploratory cross-sectional design of this study, no causality inferences could be made. Second, we used secondhand data and were unable to capture certain information; the lack of data on the personality traits and preferences of children does not support further in-depth analysis of the role of these aspects in terms of how CCT affected parents' HAI. Third, the results obtained in this study may be related to its particular cultural and environmental context. Considering that filial piety is a key virtue in Chinese culture and is considered the bedrock of the social care and health system to protect and support older people in China (21). It is likely that more latent factors remain to be found. Therefore, caution should be taken when extending the present findings to address the needs of middle-aged and older parents across diverse cultural and ethnic contexts.

## Conclusions and Implications

In conclusion, this study highlights the importance of evaluating HAI through an approach that considers both CCT and CSF. CCT and CSF had significant predictive effects on HAI. CSF both mediated and moderated the influence of CCT on HAI. These findings indicate that improving CCT and CSF may meaningfully improve HAI. The public health implications of this study are that it provides the basis for intervention strategies for a family centered program to promote healthy aging for older Chinese parents, especially for those who live in rural China.

## Data Availability Statement

The original contributions presented in the study are included in the article/Supplementary Material, further inquiries can be directed to the corresponding author/s.

## Ethics Statement

The studies involving human participants were reviewed and approved by the Biomedical Ethics Review Committee of Peking University approved CHARLS. The ethical approval number was IRB00001052-11015. The patients/participants provided their written informed consent to participate in this study.

## Author Contributions

JC contributed to conceptualization and writing the original draft preparation. YZ contributed to data curation and writing the original draft preparation. WH contributed to software and validation. JY contribute to data curation. DX contributed to resources, supervision, and funding acquisition. HL contributed to writing, reviewing, and editing. All authors contributed to the article and approved the submitted version.

## Funding

The project has received support from the Guangdong Basic and Applied Basic Research Foundation (2021A1515110743), the Swiss Agency for Development and Cooperation (Grant#81067392), and the Medical Scientific Research Foundation of Guangdong Province (Grant#A2020420). The funders had no role in the study design, data collection, data analysis, data interpretation, or writing of the report.

## Author Disclaimer

The corresponding author had full access to all the data in the study and had the final responsibility for the decision to submit for publication.

## Conflict of Interest

The authors declare that the research was conducted in the absence of any commercial or financial relationships that could be construed as a potential conflict of interest.

## Publisher's Note

All claims expressed in this article are solely those of the authors and do not necessarily represent those of their affiliated organizations, or those of the publisher, the editors and the reviewers. Any product that may be evaluated in this article, or claim that may be made by its manufacturer, is not guaranteed or endorsed by the publisher.
