# Supplementary material for: How Healthy Aging and Contact With Children Are Associated With Satisfaction in Middle-Aged and Older Parents in China: A Mediation Analysis
Source: Front Public Health. 2022 Mar 14;10:836558. doi: 10.3389/fpubh.2022.836558 (PMC8963940; doi:10.3389/fpubh.2022.836558)
Supplement: Supplementary file 1 [file Data_Sheet_1.docx]

Supplementary Material

**Supplementary Table 1** Selection of Items to Calculate Healthy Aging Index.

**Supplementary Table 2** Variable description.

**Supplementary Table 3** The Selection Process of Covariates: Step 1 - Analyzing The Relationship Between the Covariate and Y (Y=Healthy aging) One by One.

**Supplementary Table 4** The Selection Process of Covariates: Step 2 - Covariates Were Introduced into The Basic Model and Removed from the Complete Model to Observe the Change of the Regression Coefficient of X (X=Weekly Contact with Children).

**Supplementary Table 5** The Selection Process of Covariates: Step 2 - Covariates Were Introduced into The Basic Model and Removed from The Complete Model to Observe the Change of the Regression Coefficient of X (X=Satisfaction).

**Supplementary Table 6** Demographic Characteristics of Parents Aged 45 and Older Who Are contacted with Their Adult Children and Did Not.

**Supplementary Table 7** Gender and Residence Subgroup Analysis of Multinomial Logistic Regression Model for Contact with Children Associated with Satisfaction and Healthy Aging in Parents Aged 45 and Older, [RRR (95% CI)].

**Supplementary Table 8** Gender and Residence Subgroup Analysis of Multinomial Logistic Regression Model for Satisfaction Associated with Healthy Aging in Parents Aged 45 and Older, [RRR (95% CI)].

**Supplementary Table 9** Mediation analysis for Healthy Aging Associated with Contact with Children Mediated by Satisfaction in Parents Aged 45 and Older.

**Supplementary Table 1** Selection of Items to Calculate Healthy Aging Index.

| **Definition or code** |
| --- |
| 1. Ever had memory problem. |
| 2. Have difficulty running or jogging about 1 km. |
| 3. Have difficulty running or jogging about 1 km. |
| 4. Have difficulty walking 100 meters. |
| 5. Sleeping problems. |
| 6. Self-reported pain. |
| 7. Have Difficulty controlling urination and defecation. |
| 8. Hearing problem. |
| 9. Difficulties in eye sight using glasses or corrective lens as usual. |
| 10. Difficulties for seeing things at a distance, using glasses or corrective lens as usual. |
| 11. Difficulties for seeing things up close, using glasses or corrective lens as usual. |
| 12. Hearing status (with a hearing aid). |
| 13. Difficulty getting up from chair after sitting for long periods. |
| 14. Difficulty climbing several flights of stairs without resting. |
| 15. Difficulty stooping, kneeling, or crouching. |
| 16. Difficulty reaching or extending your arms. |
| 17. Difficulty lifting or carrying weights over 5 kg. |
| 18. Difficulty picking up a small coin from a table. |
| 19. Difficulty with dressing |
| 20. Difficulty with bathing or showering. |
| 21. Difficulty with eating |
| 22. Difficulty with getting out of bed and walking. |
| 23. Difficulty using the toilet, including getting up or down. |
| 24. Difficulty managing money. |
| 25. Difficulty taking medications. |
| 26. Difficulty shopping for groceries. |
| 27. Difficulty preparing a hot meal. |
| 28. Difficulty cleaning house. |
| 29. Speech problem . |
| 30. Immediate recall (Low – Lower than the 25th percentile of the distribution; Moderate or high – Higher than the 25th percentile of the distribution). |
| 31. Delayed recall (Low – Lower than the 25th percentile of the distribution; Moderate or high – Higher than the 25th percentile of the distribution). |
| 32. Numeracy (Low – Lower than the 25th percentile of the distribution; Moderate or high – Higher than the 25th percentile of the distribution). |

**Supplementary Table 2** Variable Description.

| **Variable name** | **Definition or code** |
| --- | --- |
| **Dependent variable** |  |
| HAI | healthy aging index score, 1=Q1 (“very low” of HAI); 2=Q2 (“low”); 3=Q3 (“moderate”); 4=Q4 (“high”);5 =Q5 (“very high”) |
| **Mediation variable** |  |
| CSF | Satisfaction with the relationship with children, 1=Not satisfied (Not very satisfied, Not at all satisfied); 2=Somewhat satisfied; 3=Satisfied (Completely satisfied, Very satisfied). |
| **Independent variables** |  |
| Daily CCT | Contact with children daily, 0=contact in person/phone more than one day apart; 1=contact in person/phone daily. |
| Weekly CCT | Contact with children weekly, 0=contact in person/phone more than one week apart; 1=contact in person/phone at least once a week. |
| Monthly CCT | Contact with children monthy, 0=contact in person/phone more than one month apart; 1= contact in person/phone at least one month. |
| Twice a year CCT | Contact with children twice a year, 0=contact in person/phone more than half-year apart; 1= contact in person/phone at least half-year. |
| **Covariates** |  |
| Age | -- |
| Gender | 1=male; 2=female. |
| Marital status | 1=married or partnered; 2=separated, divorced or widowed. |
| Hukou status | Hukou indicates the respondent’s hukou place and is a special identifier in China. Hukou status affects many aspects of life in China such as buying a house, buying a car, children’s school enrollment and other welfare. 1=Agricultural hukou; 2=Non-agricultural hukou; 3=Unified residence hukou or do not have hukou. |
| Residence | Residence indicates the household living region and is defined by  National Bureau of Statistics of the People's Republic of China. 1=rural; 2=urban. |
| Education levels | Education level is a simplified version of 1997 International Standard Classification of Education (ISCED-97) codes. 1=Less than lower secondary education; 2=Upper secondary & vocational training; 3=Tertiary education. |
| Public health insurance coverage | A code of 0 indicates that the respondent is not covered by any public health insurance plan. A code of 1 indicates that the respondent is covered by at least one type of public health insurance plan, including Urban Employee Medical Insurance, Urban Resident Medical Insurance, New Cooperative Medical Insurance, Urban and Rural Resident Medical Insurance, Government Medical Insurance, Medical Aid or other government insurance plan. |
| Current work status | Current work status indicates whether the respondent engaged in any work in the past year. Participants were coded as 1 if they engaged in agricultural work, non-agricultural employed work, non-agricultural self-employment work, or non-agricultural family business work per the labor force status of each wave respectively. And this variable was assigned 0 if the respondent is unemployed, retired, or never worked per the labor force status of each wave respectively. |
| Smoking | It indicates the respondent’s current smoking habit. 0=never smoke; 1=ever smoke but quit now; 2=still smoking now. |
| Alcohol intake | Alcohol intake indicates whether the respondent has had an alcoholic beverage in the last 12 months. A code of 0 indicates that the respondent reports not having any alcoholic beverage in the last 12 months. A code of 1 indicates that the respondent reports having had an alcoholic beverage in the last 12 months. |
| Self-report of health | 1=good, 2=fair, 3=poor. |
| Chronic diseases | 0=no, 1=yes, 2=morbidity. |
| Live near children | It is coded as 1 if any child co-resides or any non-co-resides child lives in the same city or county as the respondent. It is coded as 0 if they have living children but none of their children co-reside nor live in the same city or county as the respondent. |
| Number of alive children | 1=one; 2=two; 3=three; 4= four and over. |
| Gave money to children | Gave financial support to children. 0=no; 1=yes. |
| Received money from children | Received financial support from children. 0=no; 1=yes. |
| Household per capita consumption | Household per capita consumption is calculated by taking total household consumption divided by the number of people in the household. The amount of total household consumption as aggregated from all consumption activities: food consumption in last week, non-food in the past 30 days, and other non-food consumption in the past year. |

**Supplementary Table 3** The Selection Process of Covariates: Step 1 - Analyzing The Relationship Between the Covariate and Y (Y=Healthy aging) One by One.

| Covariates | term | beta | Se. | 95%CI Low | 95%CI Upp | *P*-value |
| --- | --- | --- | --- | --- | --- | --- |
| Marital status | Divorced or widowed | -0.0490 | 0.0394 | -0.1262 | 0.0282 | 0.2136 |
| Hukou status | Non-agricultural | 0.3677 | 0.0360 | 0.2972 | 0.4382 | <0.0001 |
|  | Other | 1.5894 | 1.3147 | -0.9875 | 4.1662 | 0.2267 |
| Residence | Urban | 0.3551 | 0.0284 | 0.2995 | 0.4107 | <0.0001 |
| Education levels | Upper secondary &  vocational training | 0.5235 | 0.0467 | 0.4320 | 0.6150 | <0.0001 |
|  | Tertiary education | 0.8343 | 0.1227 | 0.5937 | 1.0748 | <0.0001 |
| Public health insurance cover | Yes | 0.1969 | 0.0747 | 0.0505 | 0.3433 | 0.0084 |
| Current work status | Yes | 0.3656 | 0.0295 | 0.3078 | 0.4235 | <0.0001 |
| Smoking | Quit now | -0.3120 | 0.0482 | -0.4064 | -0.2176 | <0.0001 |
|  | Smoke now | -0.1085 | 0.0433 | -0.1934 | -0.0236 | 0.0123 |
| Alcohol intake | Yes | 0.2856 | 0.0322 | 0.2226 | 0.3487 | <0.0001 |
| Self-report of health | Fair | -0.6995 | 0.0311 | -0.7605 | -0.6385 | <0.0001 |
|  | Poor | -1.8064 | 0.0337 | -1.8724 | -1.7404 | <0.0001 |
| Chronic condition | Yes | -0.4133 | 0.0497 | -0.5108 | -0.3158 | <0.0001 |
|  | Morbidity | -1.0903 | 0.0434 | -1.1755 | -1.0052 | <0.0001 |
| Household per capita consumption | - | 0.0000 | 0.0000 | 0.0000 | 0.0000 | 0.0001 |
| Live near children | Yes | -0.0542 | 0.0273 | -0.1078 | -0.0006 | 0.0474 |
| Number of alive children | 2 | -0.2295 | 0.0482 | -0.3239 | -0.1350 | <0.0001 |
|  | 3 | -0.3878 | 0.0509 | -0.4876 | -0.2879 | <0.0001 |
|  | ≥4 | -0.5386 | 0.0544 | -0.6452 | -0.4319 | <0.0001 |
| Gave money to children | Yes | 0.2189 | 0.0290 | 0.1620 | 0.2757 | <0.0001 |
| Received money from children | Yes | -0.1018 | 0.0285 | -0.1576 | -0.0460 | 0.0004 |

**Supplementary Table 4** The Selection Process of Covariates: Step 2 - Covariates Were Introduced into The Basic Model and Removed from The Complete Model to Observe the Change of the Regression Coefficient of X (X=Weekly Contact with Children).

| Covariates | **Basic model** | **Complete model** | **Selected** |
| --- | --- | --- | --- |
| Original coefficient | 0.1839 | 0.0751 |  |
| Marital status | 0.1826 | 0.0752 |  |
| Hukou status | 0.1521* | 0.0775 | Yes |
| Residence | 0.1426* | 0.0859* | Yes |
| Education levels | 0.1522* | 0.0847* | Yes |
| Public health insurance cover | 0.1830 | 0.0754 |  |
| Current work status | 0.2054* | 0.0661* | Yes |
| Smoking | 0.1898 | 0.0733 |  |
| Alcohol intake | 0.1834 | 0.0748 |  |
| Self-report of health | 0.0939* | 0.1337* | Yes |
| Chronic condition | 0.1811 | 0.0748 |  |
| Household per capita consumption | 0.1790 | 0.0774 |  |
| Live near children | 0.1848 | 0.0753 |  |
| Number of alive children | 0.2015 | 0.0632* | Yes |
| Gave money to children | 0.1612* | 0.0803 | Yes |
| Received money from children | 0.1912 | 0.0753 |  |

* Original coefficient changed more than 10%.

**Supplementary Table 5** The Selection Process of Covariates: Step 2 - Covariates Were Introduced into The Basic Model and Removed from The Complete Model to Observe the Change of the Regression Coefficient of X (X=Satisfaction)**.**

| Covariates | **Basic model** | | **Complete model** | | **Selected** |
| --- | --- | --- | --- | --- | --- |
|  | Somewhat satisfied | Satisfied | Somewhat satisfied | Satisfied |  |
| Original coefficient | 0.4744 | 0.6639 | 0.2529 | 0.3258 |  |
| Marital status | 0.4730 | 0.6623 | 0.2525 | 0.3253 |  |
| Hukou status | 0.4565 | 0.6585 | 0.2545 | 0.3258 |  |
| Residence | 0.4524 | 0.6529 | 0.2566 | 0.3267 |  |
| Education levels | 0.4644 | 0.6619 | 0.2539 | 0.3243 |  |
| Public health insurance cover | 0.4690 | 0.6583 | 0.2579 | 0.3312 |  |
| Current work status | 0.4862 | 0.6681 | 0.2404 | 0.3127 |  |
| Smoking | 0.4747 | 0.6670 | 0.2522 | 0.3234 |  |
| Alcohol intake | 0.4741 | 0.6645 | 0.2524 | 0.3246 |  |
| Self-report of health | 0.2685* | 0.3273* | 0.3986* | 0.5686* | Yes |
| Chronic condition | 0.4251* | 0.5733* | 0.2682* | 0.3478* | Yes |
| Household per capita consumption | 0.4719 | 0.6622 | 0.2554 | 0.3284 |  |
| Live near children | 0.4731 | 0.6620 | 0.2527 | 0.3254 |  |
| Number of alive children | 0.4678 | 0.6670 | 0.2552 | 0.3247 |  |
| Gave money to children | 0.4765 | 0.6635 | 0.2500 | 0.3240 |  |
| Received money from children | 0.4846 | 0.6774 | 0.2528 | 0.3256 |  |

* Original coefficient changed more than 10%.

**Supplementary Table 6** Demographic Characteristics of Parents Aged 45 and Older Who Are contacted with Their Adult Children and Did Not.

| **Variable** | **Total** (n=9575) | **Weekly CCT** | | **t/χ^2^** | ***P-*value** |
| --- | --- | --- | --- | --- | --- |
|  |  | **Yes** (n=8073) | **No** (n=1502) |  |  |
| **Age** | 65.17 ± 8.39 | 65.09± 8.41 | 65.41 ± 8.17 | 1.333 | 0.183 |
| **gender** |  |  |  |  |  |
| Male | 4399 (45.94%) | 3690 (45.71%) | 709 (47.20%) | 1.141 | 0.285 |
| Female | 5176 (54.06%) | 4383 (54.29%) | 793 (52.80%) |  |  |
| **Marital status** |  |  |  | 17.172 | ＜0.001 |
| Married | 8016 (83.72%) | 6813 (84.39%) | 1203 (80.09%) |  |  |
| Divorced or widowed | 1559 (16.28%) | 1260 (15.61%) | 299 (19.91%) |  |  |
| **Hukou status** |  |  |  | 69.446 | ＜0.001 |
| Agricultural | 7953 (83.07%) | 6595 (81.69%) | 1358 (90.47%) |  |  |
| Non-agricultural | 1620 (16.92%) | 1477 (18.30%) | 143 (9.53%) |  |  |
| Other | 1 (0.01%) | 1 (0.01%) | - |  |  |
| **Living area** |  |  |  | 83.867 | ＜0.001 |
| Rural | 6356 (66.38%) | 5205 (64.47%) | 1151 (76.63%) |  |  |
| Urban | 3219 (33.62%) | 2868 (35.53%) | 351 (23.37%) |  |  |
| **Education levels** |  |  |  | 41.920 | ＜0.001 |
| Less than lower secondary | 8555 (89.35%) | 7142 (88.47%) | 1413 (94.07%) |  |  |
| Upper secondary & vocational training | 904 (9.44%) | 824 (10.21%) | 80 (5.33%) |  |  |
| Tertiary | 116 (1.21%) | 107 (1.33%) | 9 (0.60%) |  |  |
| **Public health insurance coverage** | | | | 0.567 | 0.451 |
| Not covered | 326 (3.40%) | 270 (3.34%) | 56 (3.73%) |  |  |
| Covered | 9249 (96.60%) | 7803 (96.66%) | 1446 (96.27%) |  |  |
| **Current work status** |  |  |  | 16.654 | ＜0.001 |
| Not working | 3702 (38.66%) | 3192 (39.54%) | 510 (33.95%) |  |  |
| Working | 5873 (61.34%) | 4881 (60.46%) | 992 (66.05%) |  |  |
| **Smoking** |  |  |  | 4.132 | 0.127 |
| Never | 5567 (58.14%) | 4695 (58.16%) | 872 (58.06%) |  |  |
| Quit now | 1525 (15.93%) | 1308 (16.20%) | 217 (14.45%) |  |  |
| Smoke now | 2483 (25.93%) | 2070 (25.64%) | 413 (27.50%) |  |  |
| **Alcohol intake** |  |  |  | 0.099 | 0.753 |
| Yes | 3014 (31.48%) | 2536 (31.41%) | 478 (31.82%) |  |  |
| No | 6561 (68.52%) | 5537 (68.59%) | 1024 (68.18%) |  |  |
| **Self-report of health** |  |  |  | 26.673 | ＜0.001 |
| Good | 1918 (20.04%) | 1664 (20.62%) | 254 (16.92%) |  |  |
| Fair | 4640 (48.47%) | 3946 (48.89%) | 694 (46.24%) |  |  |
| Poor | 3014 (31.49%) | 2461 (30.49%) | 553 (36.84%) |  |  |
| **Chronic condition** |  |  |  | 0.833 | 0.660 |
| No | 987 (10.31%) | 842 (10.43%) | 145 (9.65%) |  |  |
| Yes | 1881 (19.64%) | 1585 (19.63%) | 296 (19.71%) |  |  |
| Morbidity | 6707 (70.05%) | 5646 (69.94%) | 1061 (70.64%) |  |  |
| **Live near children** |  |  |  | 1.241 | 0.265 |
| No | 4814 (50.78%) | 4052 (50.54%) | 762 (52.12%) |  |  |
| Yes | 4666 (49.22%) | 3966 (49.46%) | 700 (47.88%) |  |  |
| **Number of children** |  |  |  | 22.129 | ＜0.001 |
| 1 | 939 (9.81%) | 774 (9.59%) | 165 (10.99%) |  |  |
| 2 | 3627 (37.88%) | 3033 (37.57%) | 594 (39.55%) |  |  |
| 3 | 2584 (26.99%) | 2150 (26.63%) | 434 (28.89%) |  |  |
| ≥4 | 2425 (25.33%) | 2116 (26.21%) | 309 (20.57%) |  |  |
| **Transfer to children** |  |  |  | 67.004 | ＜0.001 |
| No | 6470 (67.75%) | 5317(66.06%) | 1153 (76.82%) |  |  |
| Yes | 3080 (32.25%) | 2732 (33.94%) | 348 (23.18%) |  |  |
| **Transfer from children** |  |  |  | 24.164 | ＜0.001 |
| No | 3445 (36.11%) | 2819 (35.07%) | 626 (41.71%) |  |  |
| Yes | 6095 (63.89%) | 5220 (64.93%) | 875 (58.29%) |  |  |
| **Household per capita consumption** | 19744.68 ± 34783.65 | 20322.70 ± 36322.46 | 16636.26 ± 24708.37 | -3.767 | ＜0.001 |
| **CSF** |  |  |  | 94.991 | ＜0.001 |
| Not satisfied | 422 (4.41%) | 302 (3.74%) | 120 (7.99%) |  |  |
| Somewhat satisfied | 3651 (38.13%) | 2991 (37.05%) | 660 (43.94%) |  |  |
| Satisfied | 5502 (57.46%) | 4780 (59.21%) | 722 (48.07%) |  |  |

*Note:* CSF = Satisfaction with The Relationship with Children; CCT= contact with children. Differential distribution of demographic characteristics by contact with children were analyzed using χ ^2^ for categorical data and Kruskal-Wallis for continuous data. Values are bolded if they achieved statistical significance at *p* ≤ .05.

**Supplementary Table 7 Gender and Residence Subgroup Analysis of Multinomial Logistic Regression Model for Contact with Children Associated with Satisfaction and Healthy Aging in Parents Aged 45 and Older, [RRR (95% CI)].**

| **CCT** | **CSF** | | **HAI** | | | |
| --- | --- | --- | --- | --- | --- | --- |
|  | Somehow satisfied | Satisfied | Quintile 2 | Quintile 3 | Quintile 4 | Quintile 5 |
| **Male** |  |  |  |  |  |  |
| Daily CCT | 0.91 (0.64, 1.29) | 1.04 (0.74, 1.47) | 1.07 (0.84, 1.35) | 0.97 (0.77, 1.23) | 1.11 (0.87, 1.41) | 1.11 (0.87, 1.42) |
| Weekly CCT | 1.90 (1.30, 2.77)*** | 2.67 (1.83, 3.90)*** | 1.26 (0.93, 1.70) | 1.00 (0.74, 1.34) | 1.09 (0.80, 1.48) | 1.46 (1.05, 2.03)* |
| Monthly CCT | 3.65 (1.94, 6.86)*** | 4.60 (2.44, 8.65)*** | 1.05 (0.56, 1.96) | 1.97 (0.95, 4.09) | 1.30 (0.66, 2.57) | 1.84 (0.87, 3.88) |
| Twice a year CCT | 3.95 (1.51, 10.33)** | 7.28 (2.63, 20.18)*** | 0.82 (0.26, 2.52) | 2.14 (0.54, 8.49) | 1.16 (0.34, 3.91) | 2.13 (0.56, 8.11) |
| **Female** |  |  |  |  |  |  |
| Daily CCT | 0.98 (0.74, 1.29) | 1.09 (0.83, 1.43) | 0.96 (0.80, 1.13) | 0.95 (0.78, 1.14) | 1.04 (0.85, 1.27) | 1.08 (0.86, 1.36) |
| Weekly CCT | 1.50 (1.09, 2.06)* | 2.30 (1.68, 3.15)*** | 1.19 (0.95, 1.49) | 1.06 (0.83, 1.36) | 1.24 (0.95, 1.63) | 1.35 (0.97, 1.86) |
| Monthly CCT | 3.04 (1.71, 5.41)*** | 3.84 (2.19, 6.74)*** | 1.37 (0.84, 2.25) | 2.13 (1.15, 3.94)* | 3.18 (1.52, 6.63)** | 3.06 (1.31, 7.17)* |
| Twice a year CCT | 1.97 (0.63, 6.14) | 2.53 (0.83, 7.72) | 1.15 (0.47, 2.83) | 1.85 (0.62, 5.46) | 2.34 (0.71, 7.65) | 2.58 (0.65, 10.22) |
| **Rural** |  |  |  |  |  |  |
| Daily CCT | 0.88 (0.68, 1.14) | 0.99 (0.77, 1.28) | 0.95 (0.80, 1.12) | 0.86 (0.72, 1.02) | 1.00 (0.83, 1.20) | 0.98 (0.80, 1.20) |
| Weekly CCT | 1.60 (1.21, 2.11)*** | 2.28 (1.73, 3.00)*** | 1.24 (1.01, 1.52)* | 0.99 (0.80, 1.22) | 1.13 (0.90, 1.42) | 1.39 (1.07, 1.80)* |
| Monthly CCT | 4.16 (2.60, 6.67)*** | 4.50 (2.86, 7.08)*** | 1.49 (0.98, 2.28) | 2.78 (1.63, 4.73)*** | 2.87 (1.64, 5.03)*** | 2.75 (1.50, 5.04)** |
| Twice a year CCT | 3.25 (1.45, 7.30)** | 4.94 (2.20, 11.08)*** | 1.31 (0.61, 2.78) | 3.73 (1.35, 10.31)* | 2.28 (0.92, 5.65) | 3.49 (1.21, 10.05)* |
| **Urban** |  |  |  |  |  |  |
| Daily CCT | 1.09 (0.74, 1.60) | 1.23 (0.84, 1.81) | 1.14 (0.88, 1.47) | 1.18 (0.91, 1.52) | 1.26 (0.96, 1.65) | 1.33(1.00, 1.77) |
| Weekly CCT | 1.92 (1.18, 3.11)** | 3.12 (1.92, 5.07)*** | 1.12 (0.75, 1.66) | 1.16 (0.78, 1.73) | 1.25(0.82, 1.90) | 1.51 (0.96, 2.38) |
| Monthly CCT | 1.44 (0.47, 4.37) | 3.39 (1.06, 10.85)* | 0.50 (0.17, 1.50) | 0.66 (0.20, 2.13) | 0.44(0.14, 1.39) | 0.92(0.25, 3.47) |
| Twice a year CCT | 1.85 (0.36, 9.55) | 3.08 (0.57, 16.73) | 0.27 (0.03, 2.54) | 0.26 (0.03, 2.55) | 0.32 (0.03, 3.35) | 0.40 (0.03, 4.65) |

*Note.* *N* = 9675. RRR = relative risk ratio; 95% CI = 95% confidence intervals; CSF = Satisfaction with The Relationship with Children; CCT= contact with children; HAI = healthy aging. Pseudo R^2^ values in adjusted models for age, gender, hukou status, residence, education levels, public health insurance cover, current work status, smoking, alcohol intake, self-report of health, chronic condition, household per capita consumption, live near children, number of alive children, gave money to children, received money from children. Values are bolded if they achieved statistical significance at p ≤ .05.

* *p*＜0.05, ** *p*＜0.01, *** *p*＜0.001.

**Supplementary Table 8 Gender and Residence Subgroup Analysis of Multinomial Logistic Regression Model for Satisfaction Associated with Healthy Aging in Parents Aged 45 and Older, [RRR (95% CI)].**

| **CSF** | **HAI** | | | |
| --- | --- | --- | --- | --- |
|  | Quintile 2 | Quintile 3 | Quintile 4 | Quintile 5 |
| **Male** |  |  |  |  |
| Somewhat satisfied | 1.54 (0.94, 2.53) | 1.80 (1.08, 3.03)* | 2.33 (1.30, 4.19)** | 3.52(1.74, 7.10)*** |
| Satisfied | 1.25 (0.77, 2.05) | 1.53 (0.91, 2.55) | 2.19 (1.22, 3.90)** | 3.63 (1.81, 7.29)*** |
| **Female** |  |  |  |  |
| Somewhat satisfied | 1.34 (0.94, 1.92) | 1.32 (0.87, 2.00) | 1.86 (1.13, 3.06)* | 2.06 (1.06, 4.00)* |
| Satisfied | 1.43 (1.01, 2.03)* | 1.60(1.07, 2.41)* | 2.13 (1.31, 3.47)** | 3.10 (1.61, 5.95)*** |
| **Rural** |  |  |  |  |
| Somewhat satisfied | 1.46 (1.04, 2.05)* | 1.57 (1.06, 2.31)* | 1.97(1.26, 3.10)** | 2.83(1.54, 5.20)*** |
| Satisfied | 1.43 (1.02, 2.00)* | 1.58 (1.08, 2.32)* | 2.11 (1.35, 3.31)*** | 3.57(1.96, 6.50)*** |
| **Unban** |  |  |  |  |
| Somewhat satisfied | 1.24 (0.71, 2.16) | 1.32 (0.73, 2.37) | 1.98(1.00, 3.90)* | 2.40 (1.08, 5.35)* |
| Satisfied | 1.15 (0.66, 2.00) | 1.48 (0.83, 2.66) | 2.14 (1.08, 4.21)* | 3.08 (1.39, 6.82)** |

*Note:* RRR = relative risk ratio; 95% CI = 95% confidence intervals; CSF = Satisfaction with The Relationship with Children; HAI = healthy aging. Pseudo R^2^ values in adjusted models for age, gender, hukou status, residence, education levels, public health insurance cover, current work status, smoking, alcohol intake, self-report of health, chronic condition, household per capita consumption, live near children, number of alive children, transfer gave money to children, received money from children, weekly contact. Values are bolded if they achieved statistical significance at *p* ≤ .05.

* *p*＜0.05, ** *p*＜0.01, *** *p*＜0.001.

**Supplementary Table 9 Mediation analysis for Healthy Aging Associated with Contact with Children Mediated by Satisfaction in Parents Aged 45 and Older.**

| **CCT** | Indirect effect | Direct effect | Total effect | Z | Sobel P value | Proportion of total effect that is mediated |
| --- | --- | --- | --- | --- | --- | --- |
| Daily CCT | 0.003* | 0.020 | 0.023 | 2.060 | 0.039* | 12.30% |
| Weekly CCT | 0.014*** | 0.061 | 0.076* | 4.338 | ＜0.001*** | 19.05% |
| Monthly CCT | 0.022*** | 0.276*** | 0.299*** | 3.724 | ＜0.001*** | 7.42% |
| Twice a year CCT | 0.025** | 0.275* | 0.301* | 2.951 | 0.003* | 8.46% |

*Note.* CSF = Satisfaction with The Relationship with Children; HAI = healthy aging. Sobel-Goodman Mediation Test was used to test the hypothesis that the indirect role was equal to 0, adjusting for potential confounders (age, gender, hukou status, residence, education levels, public health insurance cover, current work status, smoking, alcohol intake, self-report of health, chronic condition, consumption, live near children, number of alive children, gave money to children, received money from children). Values are bolded if they achieved statistical significance at p ≤ .05.

* *p*＜0.05, ** *p*＜0.01, *** *p*＜0.001.
